# Distribution, dynamics and functional roles of phosphatidylserine within the cell

**DOI:** 10.1186/s12964-019-0438-z

**Published:** 2019-10-15

**Authors:** Jason G. Kay, Gregory D. Fairn

**Affiliations:** 1 Department of Oral Biology, School of Dental Medicine, University at Buffalo, Buffalo, NY 14214 USA; 2 Keenan Research Centre for Biomedical Science, St. Michael’s Hospital, Toronto, ON M5B 1W8 Canada; 3 Department of Surgery, University of Toronto, Toronto, ON M5S 1A8 Canada; 4 Department of Biochemistry, University of Toronto, Toronto, ON M5S 1A8 Canada

**Keywords:** Phosphatidylserine, Plasma membrane, Flippase, Scramblase

## Abstract

Phosphatidylserine (PtdSer), an essential constituent of eukaryotic membranes, is the most abundant anionic phospholipid in the eukaryotic cell accounting for up to 10% of the total cellular lipid. Much of what is known about PtdSer is the role exofacial PtdSer plays in apoptosis and blood clotting. However, PtdSer is generally not externally exposed in healthy cells and plays a vital role in several intracellular signaling pathways, though relatively little is known about the precise subcellular localization, transmembrane topology and intracellular dynamics of PtdSer within the cell. The recent development of new, genetically-encoded probes able to detect phosphatidylserine is leading to a more in-depth understanding of the biology of this phospholipid. This review aims to give an overview of recent developments in our understanding of the role of PtdSer in intracellular signaling events derived from the use of these recently developed methods of phosphatidylserine detection.

## Background

The ability to produce phosphatidylserine (PtdSer) is essential for mammalian survival [1], while the lack of PtdSer production in yeast leads to growth defects and an increase in other negatively charged lipids in an attempt at compensation [2, 3]. In addition, over production of PtdSer leads to the congenital disease Lenz-Majewski syndrome, characterized by the combination of sclerosing bone dysplasia, intellectual disability and distinct craniofacial, dental, cutaneous and distal-limb anomalies [4].

**Fig. 1:**
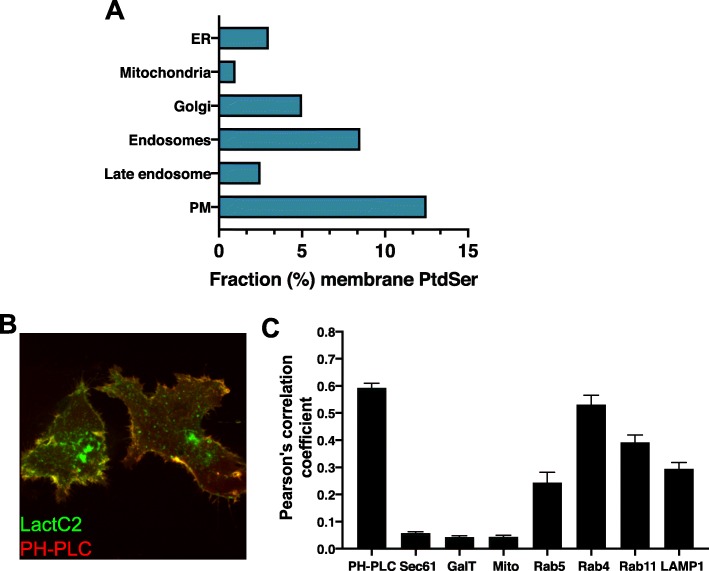
Intracellular distribution of PtdSer. **a** Relative abundance of PtdSer in membranes as mol% of total lipids throughout organelles of the cell. ER – endoplasmic reticulum, PM – plasma membrane. **b**, **c** The probe LactC2 labels cytoplasmic-facing leaflets containing PtdSer. When co-expressed with additional organellar markers (such as the plasma membrane labelling PH-PLC (**b**)) relative correlations as determined by calculation of Pearson’s correlative co-localization (**c**) can be determined as a proxy for the relative amounts of PtdSer in the cytoplasmic-facing leaflets of organelles (as first published in Hirama et al. [48]). Markers for plasma membrane (PH-PLC), ER (Sec61), Golgi (GalT), mitochondria (Mito (MitoTracker)), early endosomes (Rab5), fast and slow recycling endosomes (Rab4 and Rab11, respectively) and lysosome (LAMP1) are shown. The lack of ER and Golgi labeling by LactC2 suggests a lack of PtdSer in the cytoplasmic leaflets as discussed in the text

PtdSer has important roles in apoptosis and blood clotting, and most of what is known about PtdSer applies to these roles. However, in homeostasis PtdSer is not generally externally exposed, yet it clearly plays a vital role in healthy cells. The function of PtdSer, as with all lipids, is determined by both its concentration and sidedness in individual organellar membranes. Mitochondria associated membranes (MAMs) of the endoplasmic reticulum (ER) have high rates of PtdSer synthesis and serve as a conduit for the transfer of lipids between the ER and adjacent mitochondria [5, 6]. The bulk subcellular distribution of PtdSer results from the coordinated actions of metabolic enzymes in conjunction with vesicular and nonvesicular transport pathways, while the topology of PtdSer results from the actions of transmembrane enzymes capable of moving PtdSer between lipid bilayers; PtdSer flippases, floppases, and scramblases [7, 8]. Until relatively recently, PtdSer distribution and topology studies depended solely on the fractionation and subsequent chemical analysis of cellular organelles. These early studies highlighted PtdSer distribution throughout the cell is unbalanced (Fig. 1a), being more concentrated in the plasma membrane (PM) (~ 10–15% total lipid) with lower levels in the ER (~ 4%) and mitochondria (~ 1%), the latter of which uses PtdSer as a source of phosphatidylethanolamine (PtdEtn) (reviewed in [7, 9, 10]). The PtdSer content of less abundant organelles, including the endosomal system, has generally been less well defined because of the difficulty inherent in purifying them to homogeneity.

In addition to difference of PtdSer content amongst organelles, the unequal bilayer distribution of PtdSer at the PM has long been appreciated [11], as has the importance of movement of PtdSer from the cytoplasmic to exofacial face of the PM being involved in critical signaling events including blood clotting [12] and apoptotic cell recognition and removal by macrophages [13]. Furthermore, the PM has a net-negative charge on its cytoplasmic face [14], and consequently has an essential role in charge-based signaling events [15]. However, the contribution by PtdSer to this charge, as well as precise localization and the dynamics of PtdSer, or indeed other organelles, within whole and live cells, remains an area of active research which has recently been aided by new tools for the detection and visualization of PtdSer. In this review, we will highlight recent contributions to the understanding of PtdSer distribution and its roles within a normal cell.

### Distribution and dynamics of phosphatidylserine

The development of the PtdSer-specific LactC2 probe, based on the PtdSer-specific calcium independent discoidin-type C2 binding domain of lactadherin (also known as Milk fat globule-EGF factor 8 (MFGE8)) [16] has enabled the visualization of PtdSer in live cells (Fig. 1b-c). Indeed, the initial study using this probe showed for the first time the cytoplasmic-facing distribution of PtdSer in live cells. This initial LactC2 study underscored the importance of PtdSer in providing the negative charge of the PM, finding that cationic probes track the presence of LactC2-identified PtdSer, including in the absence of polyphosphoinositides [16]. The study also highlighted the presence of PtdSer in, and its ability to recruit charge-based protein probes to, endosomal compartments, while not being detectable in the cytoplasmic-facing cis-Golgi, ER or mitochondria. While it is possible the LactC2 probe does not have high enough sensitivity to detect the relatively low levels of PtdSer present in these organelles [9, 10], it is also possible that, like in the PM, PtdSer leaflet distribution in intracellular organelle membranes is asymmetrical [17]. Indeed, there existed significant evidence prior to the development of the LactC2 probe suggesting this is the case, at least in the ER [18–21]. This evidence has since been strengthened with additional data that does not require the biochemical isolation, and potential disruption of, this intricate tubular organelle. Using a combined light microscopy and on-section staining electron microscopy (EM) approach, the LactC2 probe was able to detect PtdSer on the luminal but not cytoplasmic facing ER membrane [22]. A modified ER-targeted LactC2 probe has also been used to successfully detect PtdSer in the ER lumen of live cells [23].

The ability of PtdSer to change membrane leaflets faces a high energy barrier, with spontaneous translocation estimated to only occur in the order of hours per single molecular translocation event [24, 25]. Three categories of proteins have been characterized that enable the trans-leaflet movement of lipids: flippases that transfer lipids to the cytosolic leaflet from the PM extracellular or organellar luminal leaflet, floppases that transfer in the opposite direction (out of the cytosolic facing leaflet), and scramblases that are bidirectional [26–28]. As the cytoplasmic leaflet of the ER is where the active site of glycerophospholipid enzymes reside [29], it has generally been thought that most glycerophospholipids in the ER are scrambled equally between leaflets to allow for proper ER membrane expansion and leaflet coupling [30, 31]. How this can be compliant with PtdSer having a polarized distribution in the lumen of the ER is unclear. However, expression of gain-of-function PtdSer synthase 1 identified from Lenz-Majewski syndrome patients does result in the appearance of cytosolic PtdSer in the ER, demonstrating that the normal mechanism(s) that restrict PtdSer to the luminal leaflet are saturable [32]. One possibility is that PtdSer, once in the luminal leaflet, is kept there through interactions with luminal proteins and/or Ca^2+^ [33]. Other non-mutually exclusive possibilities are that movement PtdSer from the cytoplasmic-facing leaflet occurs at the MAM into the mitochondria where it is used for the production of PtdEth [34], or PtdSer is removed from the cytoplasmic leaflet through non-vesicular transport by lipid transfer proteins (LTPs).

LTPs, along with vesicular trafficking, are how lipids move between cellular membranes [9, 33, 35]. Recent studies have highlighted the ability of specific LTPs, oxysterol-binding homology (Osh) proteins 6 and 7 in yeast [36, 37] and oxysterol-binding protein (OSBP)-related proteins (ORPs) 5 and 8 in mammalian cells [36, 38], to move PtdSer between membranes. The existence of these PtdSer-specific LTPs thus provide a potential mechanism for the generation and/or maintenance of the PtdSer cellular membrane gradient present in cells. Indeed, recent studies have shown that LTP-mediated transfer of PtdSer against its concentration gradient is possible through exchange with phosphatidylinositol 4-phosphate (PtdIns4P) down its concentration gradient from the PM to the ER, where the phosphatase Sac1 converts PtdIns4P to PtdIns [38, 39]. However, recent evidence suggests this exchange may be principally used to fine tune the PM levels of PtdIns4P and PtdIns(4,5)P_2_ rather than be responsible for bulk movement of PtdSer into the PM [39, 40]. There is also compelling evidence for the importance of vesicular trafficking in being the major route for PtdSer trafficking and concentration within the PM. For example, in yeast with temperature-sensitive mutations in secretory proteins Sec6 and Sec1, the polarization of PtdSer in the PM normally seen at a forming bud is inhibited and PtdSer instead accumulates on the vesicle that are prevented from fusing with the PM [2]. Additionally, endosomal recycling is important in the maintenance of high PtdSer levels, with inhibition causing a redistribution of PtdSer throughout the endosomal system in yeast [41]. Similarly, disrupting LTP function in mammalian cells has been found to result in slightly altered, but not disrupted, cellular membrane PtdSer distribution [38, 39]. Furthermore, Snx4, a member of the sorting nexin family of proteins involved in endosomal cargo sorting and recycling [42] that is specifically involved in recycling of Snc1 in yeast [43] and transferrin receptor in mammalian cells [44] has recently been implicated in leading to the modification of endosomal PtdSer levels [41].

Thus, while nonvesicular lipid transport, mediated by LTPs, play an important role, vesicular trafficking appears to be a significant contributor for maintaining the inter-membrane PtdSer gradient within the cell. Though the full molecular mechanisms of how PtdSer is segregated from other lipids remains to be fully elucidated, biochemical studies indicate a significant fraction of PtdSer in mammalian cells is enriched in PM-derived detergent-resistant, cholesterol-enriched “lipid-rafts” [45]. This biochemical data is supported by both electron microscopy analysis showing PtdSer is not homogenously distributed throughout the PM [22] and the finding that cholesterol and PtdSer co-segregate throughout subcellular compartments, being most concentrated in the PM and early endosomal compartments and relatively absent from the ER [22, 46, 47]. Further, acute changes in either affect the distribution of the other; cholesterol is required for the normal distribution of PtdSer [2, 48] and acute changes in PM levels of PtdSer alter the distribution of cholesterol [46]. Evidence is also building for the likelihood that plasma membrane outer leaflet rafts, dependent on glycersphingolipids and cholesterol [49], are coupled to inner leaflet rafts [50, 51]. The importance of PtdSer in this coupling, in both the PM and endosomal membranes, is the subject of a recent excellent review [52] so will not be further covered here.

### Roles of intracellular phosphatidylserine

**Fig. 2:**
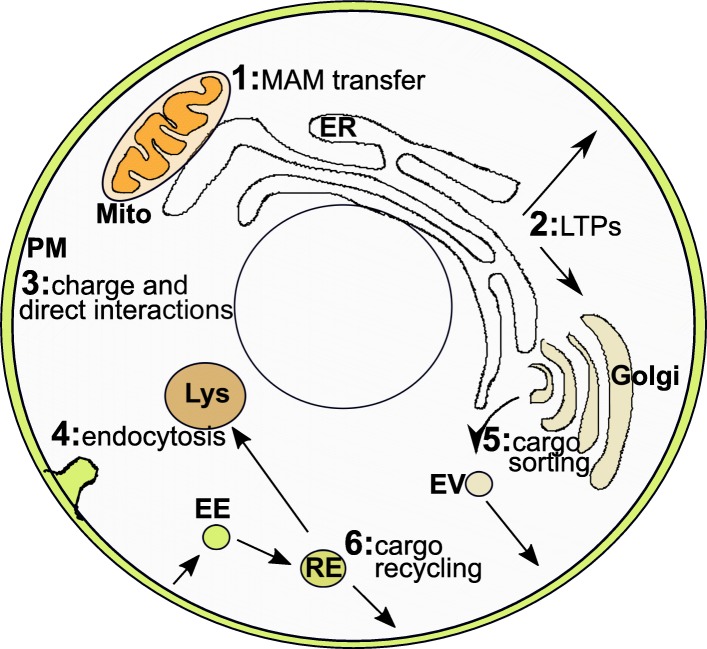
Current knowledge of roles and intracellular transport of PtdSer. PtdSer is produced in the ER, from where it is distributed throughout the cell. PtdSer can be transferred to the mitochondria through mitochondria associated membranes (MAMs) (**1**), where it is mostly converted to PtdEtn. Distribution to the PM and endosomal system can occur via traditional vesicle-mediated trafficking as well as via direct movement via PtdSer-specific lipid transfer proteins (**2**). The relative importance of both trafficking methods is currently unclear. At the PM (**3**), PtdSer is kept in the cytoplasmic-facing leaflet and is important for generating a high net-negative charge. A number of important signaling molecules are recruited to the PM through charge and/or direct PtdSer recognition binding, with PtdSer thus playing essential roles in many signaling cascades and protein localization. PtdSer also plays important roles in endocytosis (**4**), including through its curvature-inducing headgroup interactions as well as interactions with proteins required for caveolae formation. PtdSer may also play a role in Golgi function (**5**), related to cargo sorting and budding from the trans-Golgi. PtdSer also appears to be important for recycling of cargo and interaction with the recycling machinery (e.g. Evectin2, EHD1, Snx4) at the recycling endosome (**6**). These interactions with the recycling machinery also likely helps to ensure PtdSer returns to, and maintains its enrichment on, the PM while causing reduced PtdSer levels on the late endosomes and lysosomes. Mito – mitochondria, ER – endoplasmic reticulum, PM – plasma membrane, EV – exocytic vesicle, EE – endocytic vesicle, RE – recycling endosome, Lys – lysosome

As described in Background, PtdSer is essential in mammalian cells [1], while yeast lacking PtdSer are viable but have greatly reduced growth kinetics [2, 3]. As well, as PtdSer-mediated extracellular signaling, such as during blood clotting and apoptosis, has recently been reviewed [53–55], we will focus here on information regarding the roles of PtdSer within healthy non-apoptotic cells (Fig. 2).

As described, at steady state in a healthy cell PtdSer makes up to ~ 15 mol% of the total lipid in the PM. Furthermore, as it is nearly exclusively in the inner (cytoplasmic-facing) leaflet it can therefore make up to ~ 30 mol% of the lipid on this leaflet. As the major lipid with a net-negative charge, PtdSer is therefore responsible for providing much of the inner leaflet’s charge density. A significant role of PtdSer then is interacting with proteins in a non-specific charge-based manner to permit their appropriate localization within the cell (Table 1). For example, the protein kinase Src and Ras GTPase family members Rac1 and K-Ras are proteins whose membrane targeting requires a polycationic stretch in addition to lipid modifications [56, 57]. The polycationic stretch of K-Ras4B has a net charge of + 8, resulting in its localization almost exclusively at the PM. If PtdSer is removed [58], or if the net charge of this stretch is varied the resulting mutants are directed additionally to other membranes; constructs of intermediate charge (e.g., + 5) localize to endosomal membranes [16]. Similarly, Src has a polycationic stretch next to its myristoylated residue at the N-terminus with a net charge of + 5, and the kinase was found to associate not only with the PM but also extensively with PtdSer-enriched endosomal membranes [16].

**Table 1:** Examples of Intracellular Proteins Binding or Influenced by PtdSer

Protein	Brief function description
K-Ras	An early GTPase in many signal transduction pathways, for review see [77].
Rab-GTPases	A large family of proteins controlling endocytosis, cell migration, cell progression and morphology: many are discussed in main text. Exactly how many of the Rho GTPases that bind or depend on PtdSer for their localization remains uncertain.
Src	A central non-receptor tyrosine kinase with localization dependent on PtdSer, as discussed in the main text.
Protein kinase C	Enzyme family controlling other proteins through phosphorylation [78].
Akt	Activation mediates downstream responses through protein phosphorylation, binding to both PI(3,4,5)P_3_ and PtdSer [79].
Cavin1	As discussed in main text, PtdSer is required for cavin1 and caveolin1 to form stable caveolae in vivo [68]. Cavin1 forms polyhedral lattices on PtdSer- containing liposomes [80].
Evectin-2	Retrograde endosome to Golgi trafficking [72, 73]. Specific binding to PtdSer depends on the PH-domain of the protein [72, 81].
P4-ATPases	Evolutionarily conserved lipid flippases (including ATP8A1, ATP8A2, ATP9A in mammals and Drs2 in yeast) [8]. Some are specific for PtdSer [82] and as discussed in main text, a number are important for endosomal trafficking [76, 83–86], with recent reviews providing more detail [33].

Further evidence of the importance for PtdSer in charge-based protein distributions has been observed with the phagocytic process. When pathogens cause a depletion of PtdSer from phagosomes, Src is also lost [59]. In other instances, such charged motifs are not sufficient to direct proteins to a membrane but nonetheless influence their targeting, likely playing a complementary role [56, 60]. Evidence that this is the case comes from studies in yeast where polarized PtdSer is required for the recruitment of the signaling and polarity-regulating molecule Cdc42 to the forming bud neck; without PtdSer Cdc42 remains Golgi-associated and buds are very inefficiently formed, leading to poor growth [2]. Similarly, Cdc42 and Rho1 are dependent on PtdSer polarization for their proper localization and function in *Schizosaccharomyces pombe* [61]. In yet another example, the plant GTPase Rho of Plants (ROP) family member ROP6 doesn’t appear to require PtdSer for its PM association, but does require PtdSer to be stabilized into nanodomains within the membrane upon activation that allows proper signal transduction [62]. Whether PtdSer is required for, or can modulate, signaling of other ROP family members, all of which contain a polybasic stretch of amino acids at their C-terminus [62], remains to be seen.

Traditionally, the interactions between polycationic stretches in proteins and anionic phospholipid headgroups have been thought to be strictly charge based with little specificity. However, recent evidence challenges this assumption. For instance, K-Ras4B which contains six lysine residues adjacent to a farnesylated cysteine residue, has recently been shown to interact with PtdSer preferentially [63]. The tail region of K-Ras4B adopts a series on conformations, disordered, ordered and intermediate, with the disordered being the preferred conformation. This conformation is also able to H-bond PtdSer more effectively than the other two confirmations [63]. Conversely, other proteins such as K-RasG12V and Rac1 show no preference for PtdSer [63–65]. While these are only initial studies, the results suggest that some polybasic proteins may have a preference for PtdSer or other anionic lipids beyond simple electrostatically driven interactions.

There are also multiple lines of evidence indicating the charge of PtdSer contributes to PM curvature and is important for the formation of some forms of endocytic vesicles. For example, caveolae are bulb-shaped nanodomains (50–100 nm) of the PM that have been linked with many physiological functions, including mechanosensing and endocytic transport [66]. While caveolae have been known to be enriched for cholesterol and specific glycerosphingolipids, including GM3 [67], PtdSer has recently been identified as being required for their formation and maintenance [68]. This is likely at least partly due to the charge-based PtdSer binding of the cavin1 protein [69] which, along with caveolin1, is required for in vivo caveola formation [70]. PtdSer is also capable of causing membrane curvature and induce endocytosis upon the acute removal of cholesterol, again a consequence of the charged headgroup of PtdSer [48]. It is likely that cholesterol, which makes up ~ 40 mol% of PM lipids [10], helps to keep the PtdSer headgroup charge density on the inner leaflet low enough to not induce spontaneous curvature. However, once cholesterol is removed the distance between phospholipid headgroups is decreased, resulting in high spontaneous curvature capable of forming endocytic tubules [48, 71]. Indeed, increasing PtdSer levels on the inner leaflet of the PM above homeostatic levels (and therefore charge density) without concomitant cholesterol removal is also sufficient to increase formation of endocytic vesicles [48]. It is tempting to speculate that the cavin and caveolin proteins are taking advantage of this curvature-inducing property of PtdSer to induce caveolae. Thus, while cholesterol appears important for PtdSer cellular localization, it also appears to be important for modulation of PtdSer spacing and membrane curvature induction. This intimate relationship with cholesterol likely plays important roles in other PtdSer function as well, as suggested by PtdSer dynamics and interactions with caveolae [68] and signaling proteins [2, 59, 62].

The understanding of the role of PtdSer in internal membranes remains even less clear than the roles at the PM. Similar to the plasma membrane, recycling endosomes are rich in PtdSer [72] and recent work has demonstrated that PtdSer supports a variety of functions in these endosomes. The endosomal protein Evectin-2 contains a pleckstrin homology domain that binds to PtdSer rather than phosphoinositides [72]. Depletion of Evectin-2 or decreasing the availability of PtdSer prevents the movement of cholera toxin from the recycling endosome to the Golgi. Similarly, depletion of Evectin-2 and a reduction of PtdSer levels results in an inability of Golgi proteins (e.g. TGN38) to be retrieved from endosomes [72, 73]. In addition to the presence of PtdSer on the cytosolic leaflet of recycling endosomes, PtdSer flippases (e.g., ATP8A1, ATP8A2) are also required to support trafficking events. One critical effector downstream of flipped PtdSer is the Eps15 homology domain-containing protein-1 (EHD1), an ATPase with dynamin-like activity and a role in membrane remodeling required for the retrograde transport of Shiga toxin to the Golgi [74, 75]. Curiously, PtdSer, Evectin-2 and ATP8A1 have all recently been implicated as regulators of Yes-associated protein (YAP) signaling and cell proliferation [76]. ATP8A1 knockdown results in the activation of Lats, which in turn phosphorylates YAP and prevents its translocation into the nucleus. Silencing of Evectin-2 results in a decrease of Nedd4-mediated ubiquitination of Lats1, resulting in increased levels that also result in increased phosphorylation and inactivation of YAP. These studies raise several questions regarding how PtdSer and its flipping in recycling endosomes are controlling these effectors. Additionally, since recycling endosomes receive a lot of incoming membrane from the asymmetric plasma membrane, it is unclear where the luminal leaflet PtdSer is coming from to serve as a substrate for the flippases. Much is still to be learned regarding the cell physiology of PtdSer and we anticipate that the same biophysical properties PtdSer imposes on the plasma membrane will hold in endosomes and the *trans-*Golgi.

## Conclusions

It is becoming clear through recent studies that the essential phospholipid PtdSer is important for many intracellular processes in addition to its well-characterized roles in apoptosis and blood clotting. This advancement of our understanding of the intracellular roles for PtdSer has been fueled in part by the recent development of new probes to detect PtdSer. However, as described, our knowledge of the normal roles for PtdSer in both signaling and cellular trafficking within the normal cell is still developing and many details remain to be discovered.

## Data Availability

Not applicable.

## Abbreviations

EHD1: Eps15 homology domain-containing protein-1
ER: endoplasmic reticulum
LTP: lipid transfer protein
ORPs: oxysterol-binding protein-related proteins
Osh: oxysterol-binding homology
PM: plasma membrane
PtdSer: phosphatidylserine
ROP: Rho of Plants
YAP: Yes-associated protein
